# Crystal structure of the solid solution (Sr_1.65_Pb_0.35_)Al_6_O_11_

**DOI:** 10.1107/S1600536814010216

**Published:** 2014-08-01

**Authors:** Matthias Weil

**Affiliations:** a Institute for Chemical Technologies and Analytics, Division of Structural Chemistry, Vienna University of Technology, Getreidemarkt 9/164-SC, A-1060 Vienna, Austria

**Keywords:** crystal structure, solid solution, aluminate framework

## Abstract

The title compound, di(strontium/lead) hexa­aluminate, is a member of the solid solution series (Sr_2-*x*_Pb_*x*_)Al_6_O_11_. It contains two statistically occupied *M* ^2+^ (*M* = Sr, Pb) sites [both with site symmetries ..*m*; Sr:Pb occupancy ratios = 0.756 (2):0.244 (2) and 0.8968 (19):0.1032 (19)] that are located in the voids of an aluminate framework. The *M* ^2+^ sites are surrounded by six and seven O atoms, respectively, if a cut-off *M*—O distance of 3 Å is chosen, resulting in considerably distorted *M*O_*x*_ polyhedra. The aluminate framework consists of three AlO_6_ octa­hedra (two with point-group symmetries ..2/*m* and one with ..2) sharing edges to form partially filled layers extending parallel to (100) and located at *x* = 0, 0.5. Adjacent AlO_6_ layers are linked by a network made up from two crystallographically different AlO_4_ tetra­hedra by sharing corners.

## Related literature   

The title compound was obtained during experiments to prepare the strontium analogue of the lead calcium aluminate PbCa_2_Al_8_O_15_ (Artner & Weil, 2012 ▶). For another member of the isotypic solid solution series (Sr_2-*x*_Pb_*x*_)Al_6_O_11_ with a different Sr:Pb ratio, see: Plötz & Müller-Buschbaum (1982 ▶).

## Experimental   

### Crystal data  

(Sr_1.65_Pb_0.35_)Al_6_O_11_
*M* *_r_* = 555.27
Orthorhombic, 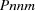

*a* = 22.0299 (4) Å
*b* = 4.8802 (1) Å
*c* = 8.3995 (2) Å
*V* = 903.03 (3) Å^3^
*Z* = 4
Mo *K*α radiation
μ = 16.94 mm^−1^
*T* = 296 K
0.12 × 0.05 × 0.05 mm

### Data collection  

Bruker SMART CCD diffractometer
Absorption correction: multi-scan (*SADABS*; Bruker, 2008 ▶) *T*_min_ = 0.236, *T*_max_ = 0.485
10434 measured reflections
2509 independent reflections
2190 reflections with *I* > 2σ(*I*)
*R*_int_ = 0.042

### Refinement  

*R*[*F* ^2^ > 2σ(*F* ^2^)] = 0.027
*wR*(*F* ^2^) = 0.061
*S* = 1.05
2509 reflections
101 parameters
Δρ_max_ = 1.12 e Å^−3^
Δρ_min_ = −1.51 e Å^−3^

### 

Data collection: *SMART* (Bruker, 2008 ▶); cell refinement: *SAINT* (Bruker, 2008 ▶); data reduction: *SAINT*; program(s) used to solve structure: *SHELXS97* (Sheldrick, 2008 ▶); program(s) used to refine structure: *SHELXL97* (Sheldrick, 2008 ▶); molecular graphics: *ATOMS for Windows* (Dowty, 2006 ▶); software used to prepare material for publication: *publCIF* (Westrip, 2010 ▶).

## Supplementary Material

Crystal structure: contains datablock(s) I, global. DOI: 10.1107/S1600536814010216/hb0009sup1.cif

Structure factors: contains datablock(s) I. DOI: 10.1107/S1600536814010216/hb0009Isup2.hkl

**Fig. 1.**
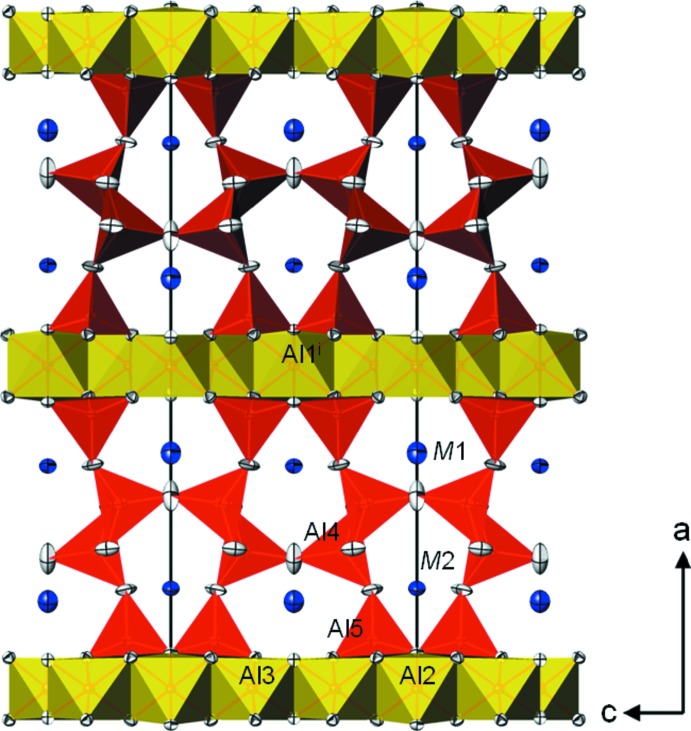
The crystal structure of (Sr1.65Pb0.35)Al6O11 in a projection along [010]. AlO6 octahedra are yellow, AlO4 octahedra are red. Displacement ellipsoids are drawn at the 97% probability level. Bonds to the statistically occupied M2+ sites (M = Sr, Pb) were omitted for clarity. [Symmetry code: i) x + 1/2, y, z.]

Click here for additional data file.1.65 0.35 6 11 6 4 M 2+ M x y z . DOI: 10.1107/S1600536814010216/hb0009fig1.tif
The crystal structure of (Sr_1.65_Pb_0.35_)Al_6_O_11_ in a projection along [010]. AlO_6_ octa­hedra are yellow, AlO_4_ octa­hedra are red. Displacement ellipsoids are drawn at the 97% probability level. Bonds to the statistically occupied *M* ^2+^ sites (*M* = Sr, Pb) were omitted for clarity. [Symmetry code: i) *x* + 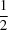, *y*, *z*.]

**Fig. 2.**
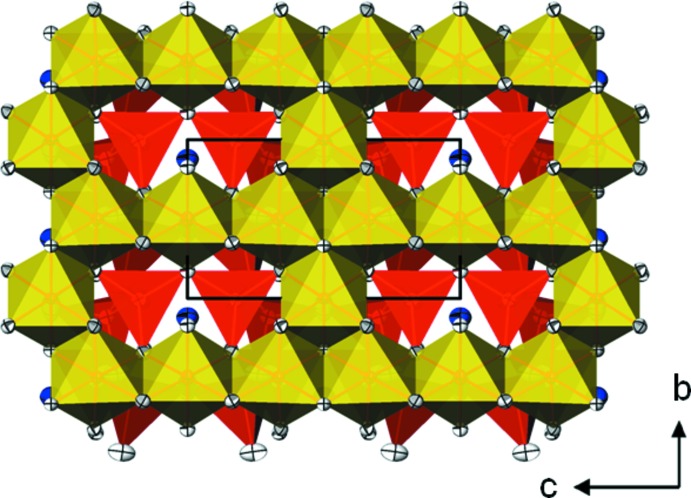
The crystal structure of (Sr1.65Pb0.35)Al6O11 in a projection along [100] showing one layer of edge-sharing AlO6 octahedra and the connecting layer of corner-sharing AlO4 tetrahedra as well as one layer of M2+ sites (M = Sr, Pb). Colour code and displacement ellipsoids are as in Fig. 1.

1.65 0.35 6 11 6 4 M 2+ M . DOI: 10.1107/S1600536814010216/hb0009fig2.tif
The crystal structure of (Sr_1.65_Pb_0.35_)Al_6_O_11_ in a projection along [100] showing one layer of edge-sharing AlO_6_ octa­hedra and the connecting layer of corner-sharing AlO_4_ tetra­hedra as well as one layer of *M* ^2+^ sites (*M* = Sr, Pb). Colour code and displacement ellipsoids are as in Fig. 1.

CCDC reference: 1004262

Additional supporting information:  crystallographic information; 3D view; checkCIF report

## supplementary crystallographic information

### S1. Experimental

A mixture of 2PbCO_3_^.^Pb(OH)_2_, SrCO_3_ and Al(OH)_3_ (molar ratio 1:6:24) was heated in a platinum crucible over a period of 24 h to 1233 K, kept at this temperature for 10 h and cooled over a period of 24 h to room temperature. A few colourless platy crystals of the title compound grew on top of a brick-red microcrystalline matrix that was not further analysed.

### S2. Refinement

Atom labels and starting parameters for refinement were taken from the isotypic compound (Sr_1.33_Pb_0.67_)Al_6_O_11_ (Plötz & Müller-Buschbaum, 1982). The two *M*^2+^ sites are statistically occupied by Pb and Sr. For each site, full occupancy was considered and the occupancy factors refined independently. The highest and lowest remaining electron densities are located 0.64 Å and 0.72 Å, respectively, from the site (Sr1/Pb1).

### Crystal data

**Table:**

(Sr_1.65_Pb_0.35_)Al_6_O_11_	*F*(000) = 1030
*M**_r_* = 555.27	*D*_x_ = 4.084 Mg m^−^^3^
Orthorhombic, *P**n**n**m*	Mo *K*α radiation, λ = 0.71073 Å
Hall symbol: -P 2 2n	Cell parameters from 3598 reflections
*a* = 22.0299 (4) Å	θ = 3.7–32.5°
*b* = 4.8802 (1) Å	µ = 16.94 mm^−^^1^
*c* = 8.3995 (2) Å	*T* = 296 K
*V* = 903.03 (3) Å^3^	Plate, colourless
*Z* = 4	0.12 × 0.05 × 0.05 mm

### Data collection

**Table:**

Bruker SMART CCD diffractometer	2509 independent reflections
Radiation source: fine-focus sealed tube	2190 reflections with *I* > 2σ(*I*)
Graphite monochromator	*R*_int_ = 0.042
ω scans	θ_max_ = 37.5°, θ_min_ = 3.7°
Absorption correction: multi-scan (*SADABS*; Bruker, 2008)	*h* = −30→37
*T*_min_ = 0.236, *T*_max_ = 0.485	*k* = −6→8
10434 measured reflections	*l* = −14→9

### Refinement

**Table:**

Refinement on *F*^2^	Primary atom site location: isomorphous structure methods
Least-squares matrix: full	*w* = 1/[σ^2^(*F*_o_^2^) + (0.0244*P*)^2^ + 0.7036*P*] where *P* = (*F*_o_^2^ + 2*F*_c_^2^)/3
*R*[*F*^2^ > 2σ(*F*^2^)] = 0.027	(Δ/σ)_max_ = 0.001
*wR*(*F*^2^) = 0.061	Δρ_max_ = 1.12 e Å^−^^3^
*S* = 1.05	Δρ_min_ = −1.51 e Å^−^^3^
2509 reflections	Extinction correction: *SHELXL97* (Sheldrick, 2008), Fc^*^=kFc[1+0.001xFc^2^λ^3^/sin(2θ)]^-1/4^
101 parameters	Extinction coefficient: 0.0022 (3)
0 restraints	

### Special details

**Table:**

Geometry. All e.s.d.'s (except the e.s.d. in the dihedral angle between two l.s. planes) are estimated using the full covariance matrix. The cell e.s.d.'s are taken into account individually in the estimation of e.s.d.'s in distances, angles and torsion angles; correlations between e.s.d.'s in cell parameters are only used when they are defined by crystal symmetry. An approximate (isotropic) treatment of cell e.s.d.'s is used for estimating e.s.d.'s involving l.s. planes.
Refinement. Refinement of *F*^2^ against ALL reflections. The weighted *R*-factor *wR* and goodness of fit *S* are based on *F*^2^, conventional *R*-factors *R* are based on *F*, with *F* set to zero for negative *F*^2^. The threshold expression of *F*^2^ > σ(*F*^2^) is used only for calculating *R*-factors(gt) *etc*. and is not relevant to the choice of reflections for refinement. *R*-factors based on *F*^2^ are statistically about twice as large as those based on *F*, and *R*- factors based on ALL data will be even larger.

### Fractional atomic coordinates and isotropic or equivalent isotropic displacement parameters (Å^2^)

**Table:**

	*x*	*y*	*z*	*U*_iso_*/*U*_eq_	Occ. (<1)
Pb1	0.364795 (11)	0.12254 (4)	0.0000	0.01369 (7)	0.756 (2)
Pb2	0.155369 (11)	0.54620 (5)	0.0000	0.01112 (8)	0.8968 (19)
Sr1	0.364795 (11)	0.12254 (4)	0.0000	0.01369 (7)	0.244 (2)
Sr2	0.155369 (11)	0.54620 (5)	0.0000	0.01112 (8)	0.1032 (19)
Al1	0.0000	0.0000	0.5000	0.00496 (19)	
Al2	0.0000	0.5000	0.0000	0.00509 (19)	
Al3	0.0000	0.5000	0.66773 (9)	0.00472 (14)	
Al4	0.21348 (3)	0.11886 (11)	0.29528 (7)	0.00638 (12)	
Al5	0.07460 (3)	0.00878 (11)	0.17903 (6)	0.00472 (11)	
O1	0.05605 (9)	0.8271 (4)	0.0000	0.0055 (3)	
O2	0.29866 (14)	0.5677 (5)	0.0000	0.0197 (5)	
O3	0.45181 (6)	0.3230 (3)	0.84219 (15)	0.0060 (2)	
O4	0.34669 (7)	0.4960 (3)	0.31766 (19)	0.0118 (3)	
O5	0.45552 (9)	0.8297 (4)	0.0000	0.0055 (3)	
O6	0.95192 (6)	0.6515 (3)	0.83661 (15)	0.0062 (2)	
O7	0.28328 (7)	0.9728 (3)	0.24121 (19)	0.0117 (3)	

### Atomic displacement parameters (Å^2^)

**Table:**

	*U*^11^	*U*^22^	*U*^33^	*U*^12^	*U*^13^	*U*^23^
Pb1	0.01691 (12)	0.01109 (9)	0.01306 (9)	0.00260 (7)	0.000	0.000
Pb2	0.00748 (12)	0.01603 (12)	0.00985 (10)	0.00238 (8)	0.000	0.000
Sr1	0.01691 (12)	0.01109 (9)	0.01306 (9)	0.00260 (7)	0.000	0.000
Sr2	0.00748 (12)	0.01603 (12)	0.00985 (10)	0.00238 (8)	0.000	0.000
Al1	0.0059 (5)	0.0041 (4)	0.0049 (4)	−0.0004 (4)	0.000	0.000
Al2	0.0039 (5)	0.0070 (4)	0.0044 (4)	0.0012 (4)	0.000	0.000
Al3	0.0043 (3)	0.0048 (3)	0.0050 (3)	0.0001 (2)	0.000	0.000
Al4	0.0049 (3)	0.0067 (2)	0.0075 (2)	−0.00021 (18)	−0.00054 (18)	0.00025 (16)
Al5	0.0035 (2)	0.0052 (2)	0.0054 (2)	−0.00008 (18)	0.00009 (17)	0.00014 (16)
O1	0.0041 (8)	0.0060 (7)	0.0063 (7)	−0.0011 (6)	0.000	0.000
O2	0.0319 (15)	0.0187 (10)	0.0086 (8)	0.0063 (10)	0.000	0.000
O3	0.0058 (6)	0.0064 (5)	0.0058 (5)	0.0007 (4)	−0.0006 (4)	0.0001 (4)
O4	0.0046 (6)	0.0151 (6)	0.0157 (6)	−0.0009 (5)	−0.0023 (5)	0.0065 (5)
O5	0.0065 (8)	0.0045 (7)	0.0055 (6)	−0.0002 (6)	0.000	0.000
O6	0.0058 (6)	0.0063 (5)	0.0065 (5)	0.0013 (4)	−0.0007 (4)	0.0000 (4)
O7	0.0067 (6)	0.0087 (6)	0.0196 (7)	0.0000 (5)	−0.0011 (5)	−0.0016 (5)

### Geometric parameters (Å, º)

**Table:**

Pb1—O5^i^	2.4569 (19)	Al1—O3^vii^	1.9055 (13)
Pb1—O3^ii^	2.5275 (13)	Al1—O3^xv^	1.9055 (13)
Pb1—O3^iii^	2.5275 (13)	Al2—O6^xi^	1.8847 (13)
Pb1—O2	2.616 (3)	Al2—O6^xvi^	1.8847 (13)
Pb1—O7^iv^	2.8042 (16)	Al2—O6^xvii^	1.8847 (13)
Pb1—O7^i^	2.8042 (16)	Al2—O6^x^	1.8847 (13)
Pb1—O2^i^	3.075 (3)	Al2—O1	2.0181 (19)
Pb1—O4	3.2558 (17)	Al2—O1^xviii^	2.0181 (19)
Pb1—O4^v^	3.2558 (17)	Al3—O3^xv^	1.9021 (13)
Pb2—O1	2.582 (2)	Al3—O3^xix^	1.9021 (13)
Pb2—O7^vi^	2.5846 (16)	Al3—O5^xx^	1.9067 (14)
Pb2—O7^vii^	2.5846 (16)	Al3—O5^vi^	1.9067 (14)
Pb2—O4^viii^	2.6771 (15)	Al3—O6^xxi^	1.9186 (14)
Pb2—O4^ix^	2.6771 (15)	Al3—O6^xxii^	1.9186 (14)
Pb2—O6^x^	2.8983 (14)	Al4—O4^vi^	1.7368 (16)
Pb2—O6^xi^	2.8983 (14)	Al4—O7^i^	1.7548 (17)
Pb2—O4^vii^	3.0914 (17)	Al4—O7^vi^	1.7556 (16)
Pb2—O4^vi^	3.0914 (17)	Al4—O2^vi^	1.7581 (8)
Pb2—O2	3.158 (3)	Al5—O4^vi^	1.7352 (16)
Al1—O5^xii^	1.8841 (18)	Al5—O3^vii^	1.7433 (14)
Al1—O5^vi^	1.8841 (18)	Al5—O6^xi^	1.7627 (14)
Al1—O3^xiii^	1.9055 (13)	Al5—O1^i^	1.7929 (11)
Al1—O3^xiv^	1.9055 (13)		
			
O5^i^—Pb1—O3^ii^	66.93 (5)	O6^xvii^—Al2—O6^x^	180.0
O5^i^—Pb1—O3^iii^	66.93 (5)	O6^xi^—Al2—O1	88.08 (5)
O3^ii^—Pb1—O3^iii^	63.26 (6)	O6^xvi^—Al2—O1	91.92 (5)
O5^i^—Pb1—O2	159.41 (8)	O6^xvii^—Al2—O1	91.92 (5)
O3^ii^—Pb1—O2	95.80 (6)	O6^x^—Al2—O1	88.08 (5)
O3^iii^—Pb1—O2	95.80 (6)	O6^xi^—Al2—O1^xviii^	91.92 (5)
O5^i^—Pb1—O7^iv^	111.67 (4)	O6^xvi^—Al2—O1^xviii^	88.08 (5)
O3^ii^—Pb1—O7^iv^	164.89 (4)	O6^xvii^—Al2—O1^xviii^	88.08 (5)
O3^iii^—Pb1—O7^iv^	101.98 (4)	O6^x^—Al2—O1^xviii^	91.92 (5)
O2—Pb1—O7^iv^	81.94 (5)	O1—Al2—O1^xviii^	180.0
O5^i^—Pb1—O7^i^	111.67 (4)	O3^xv^—Al3—O3^xix^	174.98 (9)
O3^ii^—Pb1—O7^i^	101.98 (4)	O3^xv^—Al3—O5^xx^	92.40 (7)
O3^iii^—Pb1—O7^i^	164.89 (4)	O3^xix^—Al3—O5^xx^	83.88 (7)
O2—Pb1—O7^i^	81.94 (5)	O3^xv^—Al3—O5^vi^	83.88 (7)
O7^iv^—Pb1—O7^i^	92.52 (6)	O3^xix^—Al3—O5^vi^	92.40 (7)
O5^i^—Pb1—O2^i^	82.72 (7)	O5^xx^—Al3—O5^vi^	84.72 (9)
O3^ii^—Pb1—O2^i^	134.44 (5)	O3^xv^—Al3—O6^xxi^	91.22 (6)
O3^iii^—Pb1—O2^i^	134.44 (5)	O3^xix^—Al3—O6^xxi^	92.49 (6)
O2—Pb1—O2^i^	117.87 (11)	O5^xx^—Al3—O6^xxi^	176.38 (7)
O7^iv^—Pb1—O2^i^	57.79 (4)	O5^vi^—Al3—O6^xxi^	95.43 (6)
O7^i^—Pb1—O2^i^	57.79 (4)	O3^xv^—Al3—O6^xxii^	92.49 (6)
O5^i^—Pb1—O4	115.16 (3)	O3^xix^—Al3—O6^xxii^	91.22 (6)
O3^ii^—Pb1—O4	56.39 (4)	O5^xx^—Al3—O6^xxii^	95.43 (6)
O3^iii^—Pb1—O4	107.82 (4)	O5^vi^—Al3—O6^xxii^	176.38 (7)
O2—Pb1—O4	57.78 (3)	O6^xxi^—Al3—O6^xxii^	84.65 (9)
O7^iv^—Pb1—O4	131.27 (4)	O4^vi^—Al4—O7^i^	112.79 (8)
O7^i^—Pb1—O4	58.36 (4)	O4^vi^—Al4—O7^vi^	105.98 (8)
O2^i^—Pb1—O4	115.78 (3)	O7^i^—Al4—O7^vi^	108.60 (6)
O5^i^—Pb1—O4^v^	115.16 (3)	O4^vi^—Al4—O2^vi^	111.66 (11)
O3^ii^—Pb1—O4^v^	107.82 (4)	O7^i^—Al4—O2^vi^	109.19 (12)
O3^iii^—Pb1—O4^v^	56.39 (4)	O7^vi^—Al4—O2^vi^	108.46 (10)
O2—Pb1—O4^v^	57.78 (3)	O4^vi^—Al5—O3^vii^	107.59 (8)
O7^iv^—Pb1—O4^v^	58.36 (4)	O4^vi^—Al5—O6^xi^	111.47 (7)
O7^i^—Pb1—O4^v^	131.27 (4)	O3^vii^—Al5—O6^xi^	115.93 (7)
O2^i^—Pb1—O4^v^	115.78 (3)	O4^vi^—Al5—O1^i^	102.91 (8)
O4—Pb1—O4^v^	110.07 (5)	O3^vii^—Al5—O1^i^	109.03 (7)
O1—Pb2—O7^vi^	121.11 (4)	O6^xi^—Al5—O1^i^	109.09 (7)
O1—Pb2—O7^vii^	121.11 (4)	Al5^xxiii^—O1—Al5^xxiv^	114.01 (10)
O7^vi^—Pb2—O7^vii^	114.50 (7)	Al5^xxiii^—O1—Al2	122.05 (6)
O1—Pb2—O4^viii^	63.27 (5)	Al5^xxiv^—O1—Al2	122.05 (6)
O7^vi^—Pb2—O4^viii^	68.99 (5)	Al5^xxiii^—O1—Pb2	93.98 (7)
O7^vii^—Pb2—O4^viii^	127.14 (5)	Al5^xxiv^—O1—Pb2	93.98 (7)
O1—Pb2—O4^ix^	63.27 (4)	Al2—O1—Pb2	95.65 (7)
O7^vi^—Pb2—O4^ix^	127.14 (5)	Al4^ix^—O2—Al4^viii^	155.98 (17)
O7^vii^—Pb2—O4^ix^	68.99 (5)	Al4^ix^—O2—Pb1	101.69 (8)
O4^viii^—Pb2—O4^ix^	69.79 (7)	Al4^viii^—O2—Pb1	101.69 (8)
O1—Pb2—O6^x^	59.05 (4)	Al4^ix^—O2—Pb1^xxiii^	86.97 (9)
O7^vi^—Pb2—O6^x^	141.12 (4)	Al4^viii^—O2—Pb1^xxiii^	86.97 (9)
O7^vii^—Pb2—O6^x^	88.97 (4)	Pb1—O2—Pb1^xxiii^	117.87 (11)
O4^viii^—Pb2—O6^x^	122.01 (4)	Al4^ix^—O2—Pb2	81.53 (10)
O4^ix^—Pb2—O6^x^	89.33 (4)	Al4^viii^—O2—Pb2	81.53 (10)
O1—Pb2—O6^xi^	59.05 (4)	Pb1—O2—Pb2	121.94 (10)
O7^vi^—Pb2—O6^xi^	88.97 (4)	Pb1^xxiii^—O2—Pb2	120.19 (8)
O7^vii^—Pb2—O6^xi^	141.12 (4)	Al5^xxv^—O3—Al3^xiv^	125.65 (8)
O4^viii^—Pb2—O6^xi^	89.33 (4)	Al5^xxv^—O3—Al1^xix^	119.80 (7)
O4^ix^—Pb2—O6^xi^	122.01 (4)	Al3^xiv^—O3—Al1^xix^	95.46 (6)
O6^x^—Pb2—O6^xi^	56.52 (5)	Al5^xxv^—O3—Pb1^xxvi^	111.13 (7)
O1—Pb2—O4^vii^	116.66 (5)	Al3^xiv^—O3—Pb1^xxvi^	97.22 (5)
O7^vi^—Pb2—O4^vii^	107.69 (4)	Al1^xix^—O3—Pb1^xxvi^	103.48 (5)
O7^vii^—Pb2—O4^vii^	58.03 (4)	Al5^xxv^—O3—Sr1^xxvi^	111.13 (7)
O4^viii^—Pb2—O4^vii^	174.49 (4)	Al3^xiv^—O3—Sr1^xxvi^	97.22 (5)
O4^ix^—Pb2—O4^vii^	115.37 (5)	Al1^xix^—O3—Sr1^xxvi^	103.48 (5)
O6^x^—Pb2—O4^vii^	57.61 (4)	Al5^viii^—O4—Al4^viii^	139.38 (10)
O6^xi^—Pb2—O4^vii^	86.18 (4)	Al5^viii^—O4—Pb2^vi^	92.13 (6)
O1—Pb2—O4^vi^	116.66 (5)	Al4^viii^—O4—Pb2^vi^	125.63 (8)
O7^vi^—Pb2—O4^vi^	58.03 (4)	Al5^viii^—O4—Sr2^vi^	92.13 (6)
O7^vii^—Pb2—O4^vi^	107.69 (4)	Al4^viii^—O4—Sr2^vi^	125.63 (8)
O4^viii^—Pb2—O4^vi^	115.37 (5)	Al5^viii^—O4—Pb2^viii^	88.60 (6)
O4^ix^—Pb2—O4^vi^	174.49 (4)	Al4^viii^—O4—Pb2^viii^	87.68 (6)
O6^x^—Pb2—O4^vi^	86.18 (4)	Pb2^vi^—O4—Pb2^viii^	115.37 (5)
O6^xi^—Pb2—O4^vi^	57.61 (4)	Sr2^vi^—O4—Pb2^viii^	115.37 (5)
O4^vii^—Pb2—O4^vi^	59.39 (5)	Al5^viii^—O4—Pb1	84.88 (6)
O1—Pb2—O2	146.03 (6)	Al4^viii^—O4—Pb1	80.74 (6)
O7^vi^—Pb2—O2	58.80 (4)	Pb2^vi^—O4—Pb1	90.68 (5)
O7^vii^—Pb2—O2	58.80 (4)	Sr2^vi^—O4—Pb1	90.68 (5)
O4^viii^—Pb2—O2	89.41 (5)	Pb2^viii^—O4—Pb1	153.38 (5)
O4^ix^—Pb2—O2	89.41 (5)	Al1^viii^—O5—Al3^xxvii^	96.02 (7)
O6^x^—Pb2—O2	145.70 (4)	Al1^viii^—O5—Al3^viii^	96.02 (7)
O6^xi^—Pb2—O2	145.70 (4)	Al3^xxvii^—O5—Al3^viii^	95.28 (9)
O4^vii^—Pb2—O2	92.50 (5)	Al1^viii^—O5—Sr1^xxiii^	156.90 (10)
O4^vi^—Pb2—O2	92.50 (5)	Al3^xxvii^—O5—Sr1^xxiii^	99.48 (6)
O5^xii^—Al1—O5^vi^	180.0	Al3^viii^—O5—Sr1^xxiii^	99.48 (6)
O5^xii^—Al1—O3^xiii^	95.60 (6)	Al1^viii^—O5—Pb1^xxiii^	156.90 (10)
O5^vi^—Al1—O3^xiii^	84.40 (6)	Al3^xxvii^—O5—Pb1^xxiii^	99.48 (6)
O5^xii^—Al1—O3^xiv^	84.40 (6)	Al3^viii^—O5—Pb1^xxiii^	99.48 (6)
O5^vi^—Al1—O3^xiv^	95.60 (6)	Al5^xi^—O6—Al2^xxviii^	127.55 (7)
O3^xiii^—Al1—O3^xiv^	180.0	Al5^xi^—O6—Al3^xxix^	119.36 (7)
O5^xii^—Al1—O3^vii^	84.40 (6)	Al2^xxviii^—O6—Al3^xxix^	94.41 (6)
O5^vi^—Al1—O3^vii^	95.60 (6)	Al5^xi^—O6—Pb2^xi^	94.48 (6)
O3^xiii^—Al1—O3^vii^	91.84 (8)	Al2^xxviii^—O6—Pb2^xi^	89.02 (5)
O3^xiv^—Al1—O3^vii^	88.16 (8)	Al3^xxix^—O6—Pb2^xi^	132.22 (6)
O5^xii^—Al1—O3^xv^	95.60 (6)	Al4^xxiii^—O7—Al4^viii^	118.72 (9)
O5^vi^—Al1—O3^xv^	84.40 (6)	Al4^xxiii^—O7—Pb2^viii^	100.62 (7)
O3^xiii^—Al1—O3^xv^	88.16 (8)	Al4^viii^—O7—Pb2^viii^	105.19 (7)
O3^xiv^—Al1—O3^xv^	91.84 (8)	Al4^xxiii^—O7—Sr2^viii^	100.62 (7)
O3^vii^—Al1—O3^xv^	180.0	Al4^viii^—O7—Sr2^viii^	105.19 (7)
O6^xi^—Al2—O6^xvi^	180.0	Al4^xxiii^—O7—Pb1^xxiii^	129.96 (8)
O6^xi^—Al2—O6^xvii^	86.54 (8)	Al4^viii^—O7—Pb1^xxiii^	95.99 (7)
O6^xvi^—Al2—O6^xvii^	93.46 (8)	Pb2^viii^—O7—Pb1^xxiii^	103.69 (5)
O6^xi^—Al2—O6^x^	93.46 (8)	Sr2^viii^—O7—Pb1^xxiii^	103.69 (5)
O6^xvi^—Al2—O6^x^	86.54 (8)		

Symmetry codes: (i) *x*, *y*−1, *z*; (ii) *x*, *y*, −*z*+1; (iii) *x*, *y*, *z*−1; (iv) *x*, *y*−1, −*z*; (v) *x*, *y*, −*z*; (vi) −*x*+1/2, *y*−1/2, −*z*+1/2; (vii) −*x*+1/2, *y*−1/2, *z*−1/2; (viii) −*x*+1/2, *y*+1/2, −*z*+1/2; (ix) −*x*+1/2, *y*+1/2, *z*−1/2; (x) −*x*+1, −*y*+1, *z*−1; (xi) −*x*+1, −*y*+1, −*z*+1; (xii) *x*−1/2, −*y*+1/2, *z*+1/2; (xiii) *x*−1/2, −*y*+1/2, *z*−1/2; (xiv) −*x*+1/2, *y*−1/2, −*z*+3/2; (xv) *x*−1/2, −*y*+1/2, −*z*+3/2; (xvi) *x*−1, *y*, *z*−1; (xvii) *x*−1, *y*, −*z*+1; (xviii) −*x*, −*y*+1, −*z*; (xix) −*x*+1/2, *y*+1/2, −*z*+3/2; (xx) *x*−1/2, −*y*+3/2, *z*+1/2; (xxi) −*x*+1, −*y*+1, *z*; (xxii) *x*−1, *y*, *z*; (xxiii) *x*, *y*+1, *z*; (xxiv) *x*, *y*+1, −*z*; (xxv) −*x*+1/2, *y*+1/2, *z*+1/2; (xxvi) *x*, *y*, *z*+1; (xxvii) *x*+1/2, −*y*+3/2, *z*−1/2; (xxviii) *x*+1, *y*, *z*+1; (xxix) *x*+1, *y*, *z*.
