# Supplementary figures and images for: Crystal structure of the solid solution (Sr1.65Pb0.35)Al6O11
Source: Acta Crystallogr Sect E Struct Rep Online. 2014 Aug 1;70(Pt 9):i45. doi: 10.1107/S1600536814010216 (PMC4186198; doi:10.1107/S1600536814010216)

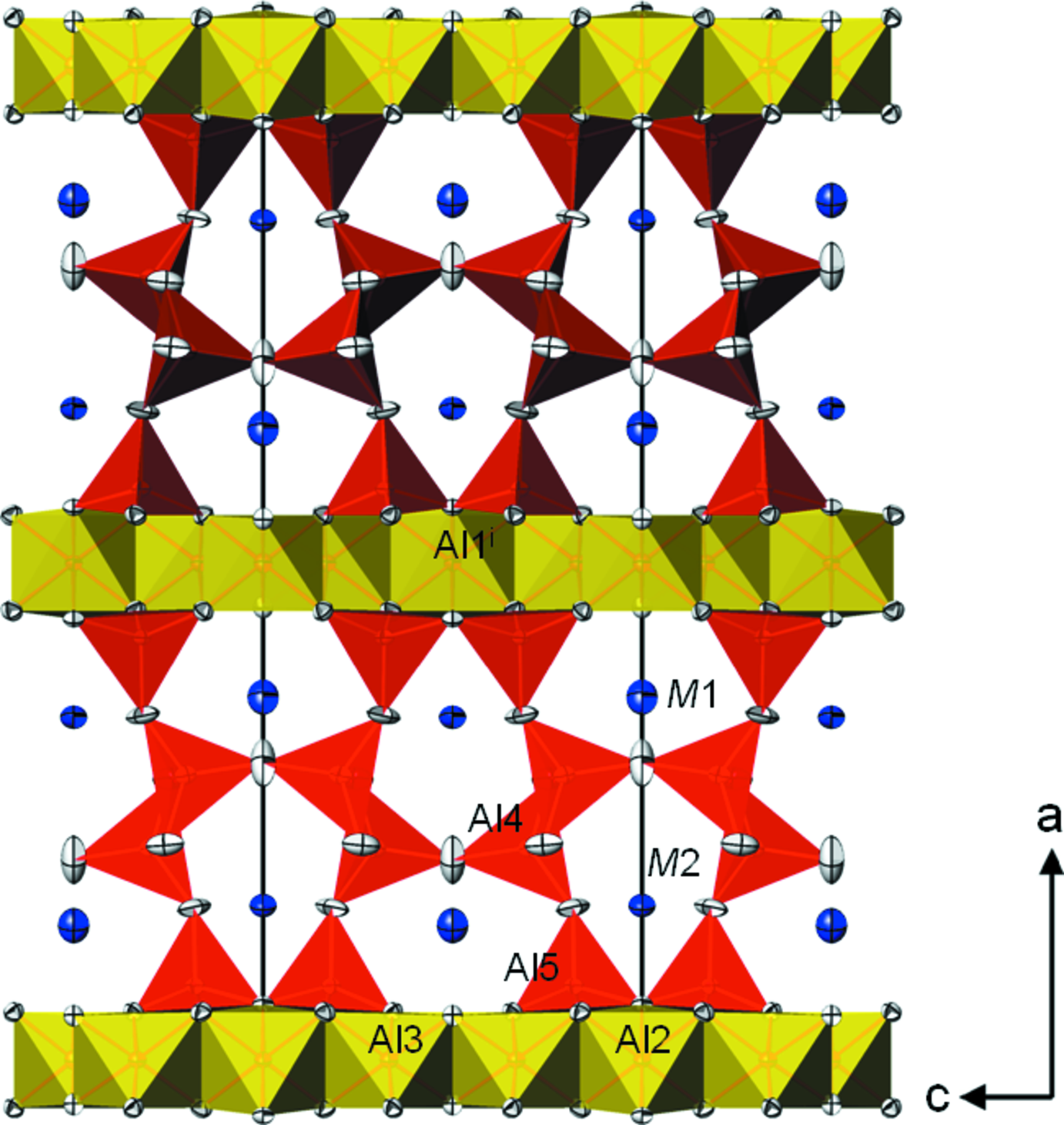

Supplement: Supplementary file 3 [file e-70-00i45-fig1.tif]

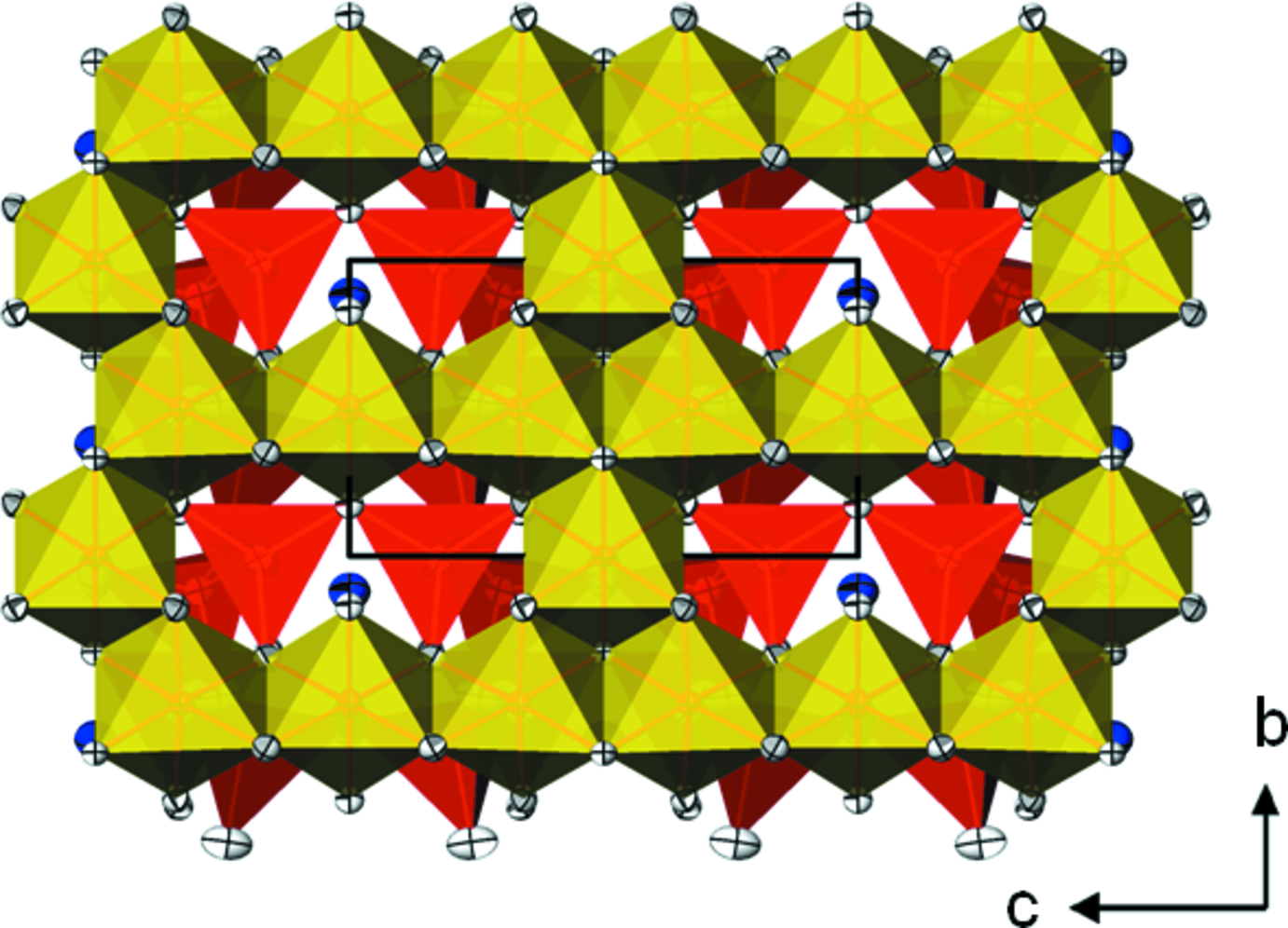

Supplement: Supplementary file 4 [file e-70-00i45-fig2.tif]
